# The Serum and Fecal Metabolomic Profiles of Growing Kittens Treated with Amoxicillin/Clavulanic Acid or Doxycycline

**DOI:** 10.3390/ani12030330

**Published:** 2022-01-29

**Authors:** Evangelia M. Stavroulaki, Jan S. Suchodolski, Rachel Pilla, Geoffrey T. Fosgate, Chi-Hsuan Sung, Jonathan Lidbury, Jörg M. Steiner, Panagiotis G. Xenoulis

**Affiliations:** 1 Clinic of Medicine, Faculty of Veterinary Science, University of Thessaly, 43131 Karditsa, Greece; pxenoulis@vet.uth.gr; 2 Gastrointestinal Laboratory, Department of Small Animal Clinical Sciences, Texas A&M University, College Station, TX 77843, USA; jsuchodolski@cvm.tamu.edu (J.S.S.); rpilla@cvm.tamu.edu (R.P.); csung@cvm.tamu.edu (C.-H.S.); jlidbury@cvm.tamu.edu (J.L.); jsteiner@cvm.tamu.edu (J.M.S.); 3 Department of Production Animal Studies, University of Pretoria, Onderstepoort, Pretoria 0110, South Africa; geoffrey.fosgate@up.ac.za

**Keywords:** antibiotics, metabolomic profile, cats

## Abstract

**Simple Summary:**

This study investigated the impact of antibiotic treatment οn the serum and fecal metabolome (the collection of all small molecules produced by the gut bacteria and the host) of young cats. Thirty 2-month-old cats with an upper respiratory tract infection were treated with either amoxicillin/clavulanic acid for 20 days or doxycycline for 28 days. In addition, another 15 control cats that did not receive antibiotics were included. Blood was collected on days 0 (before treatment), 20/28 (last day of treatment), and 300 (10 months after the end of treatment), while feces were collected on days 0, 20/28, 60, 120, and 300. Seven serum and fecal metabolites differed between cats treated with antibiotics and control cats at the end of treatment period. Ten months after treatment, no metabolites differed from healthy cats, suggesting that amoxicillin/clavulanic acid or doxycycline treatment only temporarily affects the abundance of the serum and fecal metabolome.

**Abstract:**

The long-term impact of antibiotics on the serum and fecal metabolome of kittens has not yet been investigated. Therefore, the objective of this study was to evaluate the serum and fecal metabolome of kittens with an upper respiratory tract infection (URTI) before, during, and after antibiotic treatment and compare it with that of healthy control cats. Thirty 2-month-old cats with a URTI were randomly assigned to receive either amoxicillin/clavulanic acid for 20 days or doxycycline for 28 days, and 15 cats of similar age were enrolled as controls. Fecal samples were collected on days 0, 20/28, 60, 120, and 300, while serum was collected on days 0, 20/28, and 300. Untargeted and targeted metabolomic analyses were performed on both serum and fecal samples. Seven metabolites differed significantly in antibiotic-treated cats compared to controls on day 20/28, with two differing on day 60, and two on day 120. Alterations in the pattern of serum amino acids, antioxidants, purines, and pyrimidines, as well as fecal bile acids, sterols, and fatty acids, were observed in antibiotic-treated groups that were not observed in control cats. However, the alterations caused by either amoxicillin/clavulanic acid or doxycycline of the fecal and serum metabolome were only temporary and were resolved by 10 months after their withdrawal.

## 1. Introduction

It is well known that the GI microbiome plays a central role in the host’s metabolism [1]. The communication between the host and its microbiome occurs mainly through microbially derived metabolites that act as signaling molecules. Metabolomic analysis can detect and identify a wide range of small molecules that are present in biological samples, therefore allowing the assessment of both of microbial and host-derived metabolites [2]. Metabolomic analysis can be either targeted or untargeted, meaning that it either targets predefined metabolites or unknown metabolites, respectively. Feces may be more representative of the direct microbial metabolic products produced in the gut, compared to serum metabolites, representing those metabolites that eventually enter the systemic circulation and potentially have a greater impact on the host [3,4]. 

Antibiotics are used commonly for the treatment of upper respiratory tract infections (URTI) in kittens. In humans, the extensive use of antibiotics during early life is avoided based on the risk of the development of antibiotic resistance or because of the potential long-lasting effects of antibiotics on the gastrointestinal (GI) microbiome [5,6]. Secondary metabolic perturbations can occur due to antibiotic-induced dysbiosis in infants. A combination treatment of ampicillin and gentamycin caused reduced fecal concentrations of GABA, tryptophan, and ornithine in 2-day-old infants [7]. These metabolites play an important role in neurodevelopment and intestinal contractility, while ornithine is also an energy source for enterocytes [7]. In another study in 1-week-old infants, a combination of various beta-lactam antibiotics caused reduced fecal concentrations of microbially produced antibiotic compounds [8]. Antibiotics produced by the microbiome play an important role in colonization resistance [8]. The disruption of colonization resistance is a common consequence of antibiotic therapy and might be followed by the colonization or proliferation of pathogenic bacteria, leading to persistent gastrointestinal or systemic symptoms. The percentage of the known fecal end metabolic products that are altered by antibiotic treatment has been estimated to be between 4.4% and 87% in humans [9]. Commonly perturbed metabolic pathways following antibiotic intervention relate to protein, carbohydrate, lipid, and bile acid (BA) metabolism [10]. 

No studies have previously investigated the effects of antibiotics on the serum or fecal metabolic profiles of young cats. Previous studies have focused on the taxonomical and compositional characterization of the GI microbiome in young cats [11,12,13,14,15,16,17,18]. Μore recent studies have also described the serum and fecal metabolites in cats in states of health [19,20,21,22,23] and disease [24,25,26,27,28,29], or following drug administration [30,31,32,33]. Investigating metabolic patterns under certain conditions has filled gaps in understanding cellular processes and has led to the discovery of new disease biomarkers, allowing an understanding of impaired signaling pathways in different disease states [9]. For example, increased concentrations of several amino acids, arachidonic acid, and simple sphingolipids, and reduced concentrations of indole derivatives, have been found in the feces from cats with inflammatory bowel disease and alimentary lymphoma [28]. These alterations add knowledge to the pathogenesis of feline chronic enteropathies, as well as highlight potential therapeutic strategies. In another study, Burmese cats, a breed at high risk for developing diabetes mellitus, had higher serum concentrations of tyrosine and 2-oxoisocaproic acid, which are precursors leading to insulin resistance in humans [34]. 

Antibiotic treatment has been shown to alter the abundance of serum and fecal metabolites in dogs. Tylosin and metronidazole constitute the antibiotics of choice in dogs with antibiotic-responsive enteropathy, although their effects on the microbiome and secondary metabolites suggest the maintenance of GI dysbiosis. Metronidazole administration in dogs decreased fecal vitamins, antioxidants, secondary BAs, and increased oxidative stress molecules [35,36]. A combination therapy with metronidazole and enrofloxacin led to alterations of various metabolic profiles, including short chain fatty acids (SCFAs), tryptophan, and sphingolipid metabolites, as well as reductions in secondary BAs and increases in primary BAs [37]. Treatment with tylosin also caused an increase in primary BAs [38]. In cats, only the effects of clindamycin have been investigated, and long-term changes described have included a reduction in deoxycholic acid, a secondary BA, 2 years after antibiotic withdrawal [31,32]. 

Although the mode of action of each antibiotic has been extensively studied, there is new evidence from metabolomic studies that the secondary metabolites produced during antibiotic intervention play a role in antibiotic lethality [39]. Antibiotics that target the intracellular bacterial metabolism affect different microbial metabolic pathways compared to antibiotics that target the microbial cell wall or which are bacteriostatic [40]. For example, altering amino acid metabolism is a mechanism by which bacteria acquire resistance to aminoglycosides. The exogenous administration of amino acids increases the permeability of the bacterial cell membrane, which further increases the uptake of aminoglycosides and increases their efficacy [41]. Identifying the metabolic pathways by which antibiotics kill bacterial cells can be used for the identification of molecules that strengthen antibiotic lethality.

The aim of the present study was to describe the short- and long-term effects of amoxicillin/clavulanic acid or doxycycline on the serum and fecal metabolome in young cats. A secondary aim was to describe the normal age-related changes on the abundance of feline serum and fecal metabolites during the first year of life. 

## 2. Materials and Methods

### 2.1. Study Population

This was a prospective case–control study. Forty-five rescue kittens, approximately 2 months of age, were enrolled. All kittens were kept in individual cages in appropriately designed facilities of the Clinic of Medicine at the Faculty of Veterinary Science of the University of Thessaly until adoption into private homes. Prior to inclusion into the study, all kittens received the same antiparasitic treatment (fipronil, (S)-methoprene, eprinomectin, and praziquantel; Broadline, Boehringer Ingelheim) and remained on the same antiparasitic treatment monthly throughout the study period. In addition, all kittens were fed the same commercial dry cat food during the study (GEMON Cat Breeder Kitten, Monge Breeder, Turin, Italy) and were vaccinated (Purevax RCPh, Purevax Rabies, Gerolymatos International S.A., Athens, Greece) according to standard vaccination guidelines [42]. All cats were eventually adopted by the end of the study and owners signed an informed owner consent form.

All 45 kittens had clinical signs suggestive of an acute URTI, including sneezing, ocular and/or nasal discharge, and blepharospasm for less than 10 days [43]. Concurrent health conditions were documented, and kittens were excluded if these were severe enough to require hospitalization, or if they had conditions suspected to affect the GI microbiota (such as GI infections). In addition, no more than two related cats were included in the study to ensure that relatedness did not bias results. Kittens diagnosed with URTI were chosen because acute URTI is typically restricted to the upper respiratory system, and it was presumed that this disease will not affect the concentrations of serum or fecal metabolites in cats. 

### 2.2. Group Allocation and Treatments

On day 0, kittens were randomly assigned to receive either amoxicillin/clavulanic acid at a dose of 20 mg/kg q12 h for 20 days (AMC group = 15 cats) or doxycycline at a dose of 10 mg/kg q24 h for 28 days (DOX group = 15 cats), each administered orally. Randomization was performed using Microsoft Excel (2017), with odd numbers corresponding to the AMC group, whereas even numbers corresponded to the DOX group. These antibiotics were chosen based on previously published guidelines for the treatment of URTI in cats [43]. In addition, 15 kittens that had mild clinical signs of URTI did not receive antibiotics and were assigned to the control group (CON group). 

### 2.3. Sample Collection and Follow-Up Period

Blood samples were collected from the jugular vein, allowed to clot, centrifuged at 3000× *g* for 10 min, and serum was collected and placed into Eppendorf tubes and stored at −80 °C, pending analysis. For cats in the AMC group, blood samples were collected on days 0, 20 (last day of amoxicillin/clavulanic acid treatment), and 300. For cats in the DOX and CON groups, blood samples were collected on days 0, 28 (last day of doxycycline treatment), and 300.

Fecal samples were collected from the litter box within 4 h of defecation, placed into Eppendorf tubes, and kept at −80 °C until analysis. For kittens that were adopted, owners were instructed to clean the litterbox prior to the day of each scheduled fecal sample collection, collect fresh feces on the scheduled day, and ship them packed on ice by overnight courier. For cats in the AMC group, feces were collected on days 0 (baseline), 20 (last day of amoxicillin/clavulanic acid treatment), 60, 120, and 300. For cats in the DOX and CON groups, feces were collected on days 0, 28 (last day of doxycycline treatment), 60, 120, and 300.

### 2.4. Metabolomic Analysis

#### 2.4.1. Untargeted Metabolomic Analysis of Serum Samples 

Serum samples were analyzed at the West Coast Metabolomics Center (University of California, Davis, CA, USA) using a gas chromatography–time-of-flight mass spectrometry (GC-TOF MS) method. Serum aliquots were extracted with degassed acetonitrile. Internal standards C08-C30 fatty acid methyl ethers (FAMEs) were added, and the samples were derivatized with methoxyamine hydrochloride in pyridine and subsequently with N-methyl-N-trimethylsilyltrifluoroacetamide for the trimethylsilylation of acidic protons. Analytes were separated using an Agilent 6890 gas chromatograph (Santa Clara, CA, USA), and mass spectrometry was performed on a Leco Pegasus IV time-of-flight mass spectrometer (St. Joseph, MI, USA), following a published protocol [44]. Unnamed peaks were excluded from statistical analysis and peak height data were obtained and uploaded to MetaboAnalyst 4.0 (Xia Lab, McGill University, Montreal, Canada). Then, the filtered data were normalized through log transformation and Pareto scaling [45,46]. 

#### 2.4.2. Targeted Metabolomic Analysis of Fecal Samples

Lyophilized fecal samples were used to measure the concentrations of unconjugated fecal primary BAs (cholic acid (CA) and chenodeoxycholic acid (CDCA)) and secondary BAs (lithocholic acid (LCA), deoxycholic acid (DCA), and ursodeoxycholic acid (UDCA)), fatty acids (FAs), and sterols using a gas chromatography coupled with a mass spectrometry protocol, as previously described [47,48]. Fecal concentrations of BA were expressed as ng/mg of lyophilized feces, as well as percentage of total BA. Fecal concentrations of FAs and sterols were expressed as μg/mg of lyophilized feces. 

### 2.5. Statistical Analysis

The log-transformed, Pareto-scaled untargeted serum metabolites were compared among groups using two-way ANOVA, and within groups with two-way repeated measures ANOVA. Multiple post hoc comparisons were adjusted using Tukey’s tests. *p*-values for between group comparisons were adjusted with the Benjamini and Hochberg false discovery rate (FDR), and significance was set at *q* < 0.05 to account for the large number of analytes measured. *p*-values for within-group comparisons were adjusted with Bonferroni corrections and significance was set at *p* < 0.05. 

Fecal metabolites were rank transformed prior to statistical analysis due to the failure of the normality assumptions. A linear mixed model was fitted, including time, group, and the interaction between time and group as fixed effects and cat as a random effect. Multiple pairwise post hoc comparisons were Bonferroni corrected, and significance was set at *p* < 0.05. All statistics were performed with the web-based metabolomic data processing tool Metaboanalyst 5.0. (www.metaboanalyst.ca (accessed on 29 November 2021)), GraphPad Prism 9 (GraphPad Software Inc., San Diego, CA, USA), and SPSS version 23.0. Principal coordinate analysis, and hierarchical clustering heatmaps were generated using Metaboanalyst 5.0. 

## 3. Results

### 3.1. Serum Metabolomics

**Figure 1:**
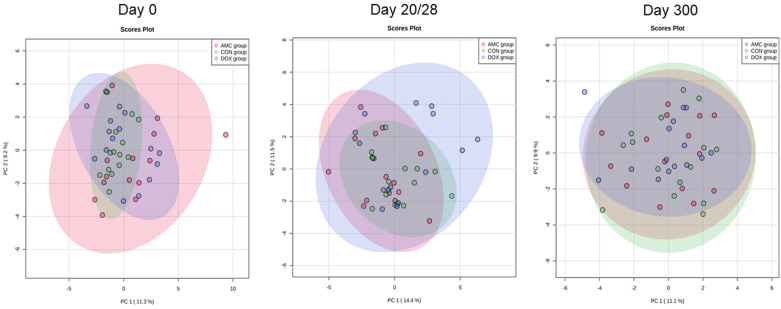
Principal coordinate analysis of serum metabolites on day 0 (before antibiotic administration), day 20/28 (last day of antibiotic treatment for the AMC and DOX groups, respectively), and day 300 for cats in the AMC, CON, and DOX groups.

A total of 182 named serum metabolites were identified, 15 of which differed significantly (*q* < 0.05) among control cats, cats treated with amoxicillin/clavulanic acid, and cats treated with doxycycline on day 0 (pre-treatment; Table 1). Principal coordinate analysis plots showed the clustering of variables based on treatment group on day 0 (Figure 1). The development of gut microbiota is mirrored in the microbially driven serum metabolites during early life, and abundances individualize around 2 months of age. A table with the raw abundances, as well as a table with the mean abundances and standard deviation of the metabolites identified in serum, are available as Table S1 and Table S2, respectively.

#### 3.1.1. Control Group

**Figure 2:**
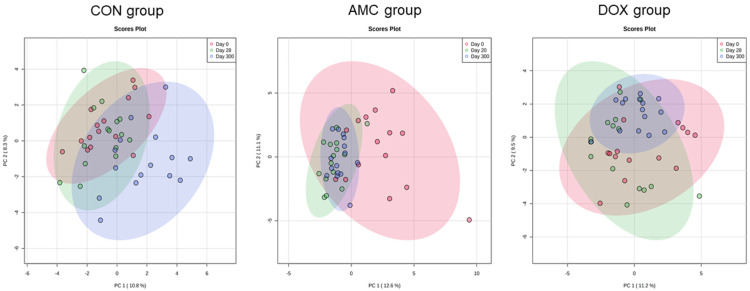
Principal coordinate analysis of serum metabolites for CON, AMC, and DOX groups on days 0, 20/28, and 300.

Table 2 shows the complete list of serum metabolites that changed in their abundances within the first year of life in control cats. None of the metabolites changed significantly from 2 to 3 months of age. Figure 2 shows how samples from days 28 (3 months of age) and 300 (1 year of age) are different from those on day 0 (2 months of age). After 3 months of age, amino acid concentrations changed with an increase in tryptophan (*p* = 0.025) and a decrease in glycine (*p* = 0.019). Decreases in metabolites classified as antioxidants (trans-4-hydroxyproline (*p* = 0.017), methionine sulfoxide (*p* = 0.013); polyamines: putrescine (*p* = 0.002), phenylethylamine (*p* = 0.037)), as well as in metabolites related to sugar metabolism (maltose (*p* = 0.008), erythritol (*p* = 0.031), arabitol (*p* = 0.008), and threonic acid (*p* = 0.038)) were also noted. 

#### 3.1.2. Amoxicillin/Clavulanic Acid Group

**Figure 3:**
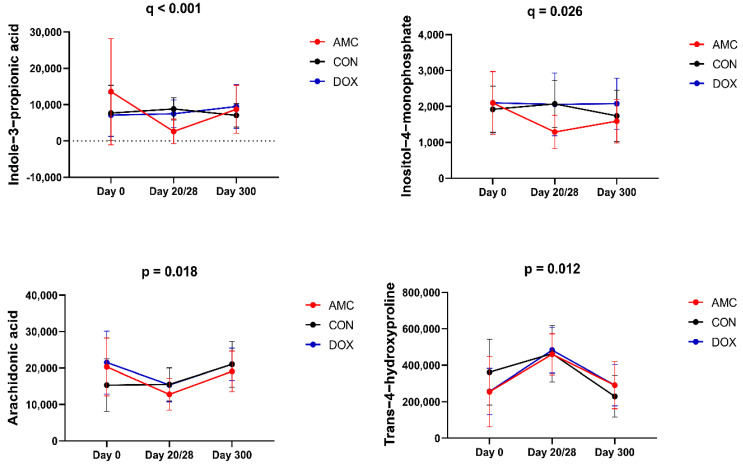
Abundance of serum metabolites that significantly differed between the AMC and CON groups on day 20/28 (indole-3-propionic acid, inositol-4-monophosphate) and from day 0 to day 20 within AMC cats (arachidonic acid, trans-4-hydroxyproline). Means and standard deviations are displayed.

Table 3 shows the complete list of serum metabolites for which the abundance changed within the first year of life in cats treated with amoxicillin/clavulanic acid. Amoxicillin/clavulanic acid caused few changes to the serum metabolites of kittens when compared to the other groups of cats. Indole-3-propionic acid (*p* < 0.001), an antioxidant, and inositol-4-monophosphate (*q* = 0.026), an inositol lipid derivative, were detected at significantly lower concentrations in AMC cats compared to CON and DOX cats on the last day of treatment (Table 1, Figure 3). 

In addition, changes in the abundance of metabolites were observed in AMC cats from 2 to 3 months of age, i.e., from day 0 to day 20, that were not observed within the control group at the same age. Aconitic acid (*p* = 0.034) and arachidonic acid (*p* = 0.018) decreased significantly after 20 days of treatment with amoxicillin/clavulanic acid. In addition, trans-4-hydroxyproline (*p* = 0.012), an antioxidant, increased by the last day of treatment (Figure 3). 

A different pattern for the abundance of metabolites was also observed in AMC cats after 3 months of age (after day 20) when compared to the CON cats (Figure 2). Within the amino acid group, threonine significantly increased (*p* = 0.025), while isoleucine decreased (*p* = 0.016) in AMC cats after 3 months of age. The serum concentrations of metabolites belonging to sugars increased, including glucose (*p* = 0.017) and fucose (*p* = 0.045). 

#### 3.1.3. Doxycycline Group

**Figure 4:**
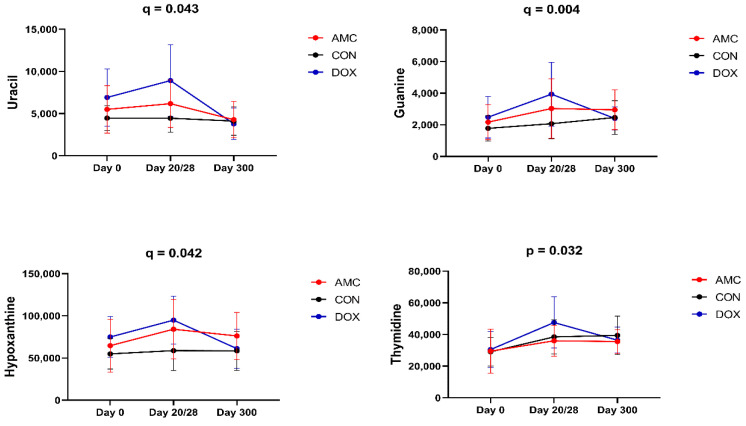
Changes in the abundance of serum metabolites that significantly differed between cats of the DOX and those of the CON group on day 20/28 (uracil, guanine, hypoxanthine) and from day 0 to day 28 within cats of the DOX group (thymidine). Means and standard deviations are displayed.

Table 4 contains the complete list of serum metabolites for which the abundance changed within the first year of life in cats treated with doxycycline. The concentrations of serum metabolites in 2-month-old cats were characterized by significant changes after 28 days of treatment with doxycycline, i.e., from 2 to 3 months of age. Concentrations of pyrimidines, including uracil (*q* = 0.043), and purines, including guanine (*q* = 0.004) and hypoxanthine (*q* = 0.042), were at significantly higher levels in DOX cats compared to CON cats on the last day of treatment (Table 1, Figure 4). 

A different pattern in the concentrations of serum metabolites in DOX cats was observed from 2 to 3 months of age when compared to CON cats at this age. Similar to AMC cats, concentrations of trans-4-hydroxyproline increased in DOX cats (*p* = 0.007) (Figure 3). In addition, thymidine increased (*p* = 0.032), while metabolites classified as vitamins and sterols, including tocopherol-alpha (*p* = 0.037) and beta-sitosterol (*p* = 0.027), respectively, decreased from 2 to 3 months of age in DOX cats. 

**Figure 5:**
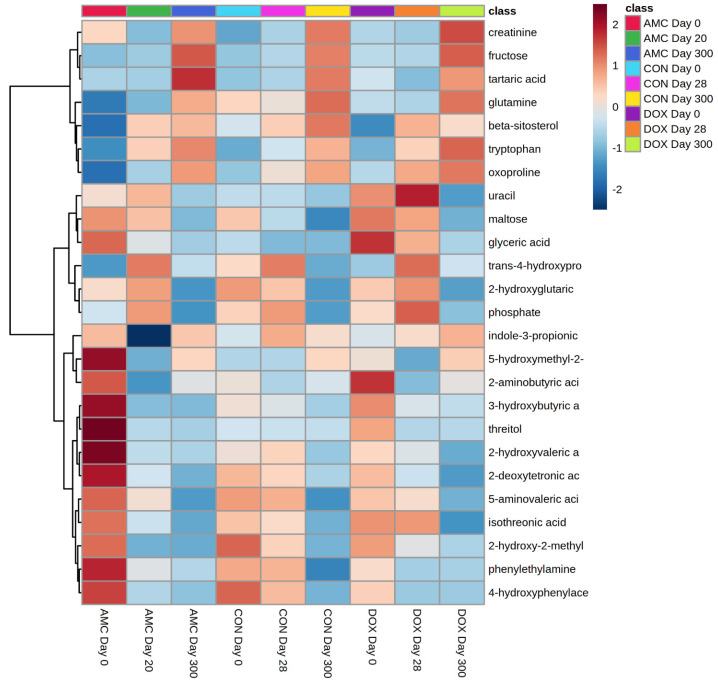
Heatmap clustering metabolites in serum of AMC, CON, and DOX cats over time (days 0, 20/28, 300). For simplicity, the figure only displays the average intensities of the top 25 metabolites that were different (*q* < 0.05) based on the ANOVA. The higher the signal intensity of a metabolite, the more intensely red the metabolite shows. The lower the signal intensity of a metabolite, the more intensely blue the metabolite shows in the heat map. Each row corresponds to a single metabolite. Each column represents the average intensity of a metabolite at a given sampling time within AMC, CON, and DOX groups, and samples are grouped by day within each treatment group along the x-axis.

The pattern of the abundance of individual metabolites was also different in DOX cats compared to CON cats after the age of 3 months (i.e., day 28) (Figure 2). Within the amino acid group, threonine (*p* < 0.001) and glutamine (*p* = 0.038) increased, while aspartic acid (*p* = 0.036) decreased by the age of 1 year. Metabolites involved in sugar metabolism were also affected, including an increase in fructose (*p* = 0.049) and a reduction in isothreonic acid (*p* = 0.002) and gluconic acid (*p* = 0.037). A heatmap, which graphically represents variations in serum metabolite signal intensities among the three groups of cats, is presented in Figure 5.

### 3.2. Fecal Metabolomics

**Figure 6:**
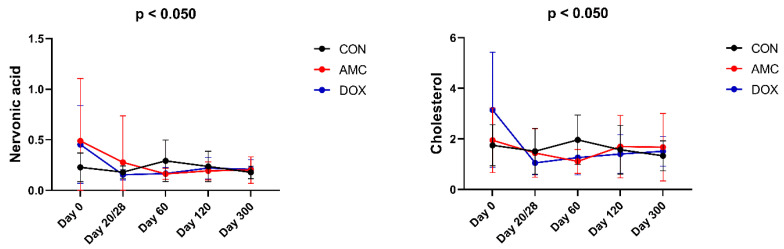
Fecal metabolites that significantly differed among AMC, DOX, and CON cats over the course of the study. Means and standard deviations are displayed.

The AMC and DOX cats had no significant differences in fecal BA concentrations compared to CON cats at any time point. There was only a tendency for decreased concentrations of secondary BAs in both antibiotic-treated groups on the last day of treatment compared to CON cats, but this did not reach statistical significance (*p* = 0.090). However, concentrations of fatty acids and sterols were significantly affected by antibiotics. Nervonic acid and cholesterol were detected at significantly lower concentrations in DOX cats compared to CON cats on the last day of treatment (nervonic acid, *p* = 0.010; cholesterol, *p* = 0.005) as well as 1 month (i.e., day 60) (nervonic acid, *p* < 0.001; cholesterol, *p* = 0.001) and 3 months (i.e., day 120) (nervonic acid, *p* = 0.037; cholesterol; *p* = 0.048) after antibiotic withdrawal. Nervonic acid concentrations were also significantly lower in AMC cats at 1 month (*p* = 0.001) and 3 months (*p* = 0.032) after antibiotic withdrawal compared to CON cats (Figure 6). A table with the raw metabolites and a table with the mean abundances and standard deviation with the metabolites identified in fecal samples are available as Table S3 and Table S4, respectively. 

#### 3.2.1. Control Group 

**Figure 7:**
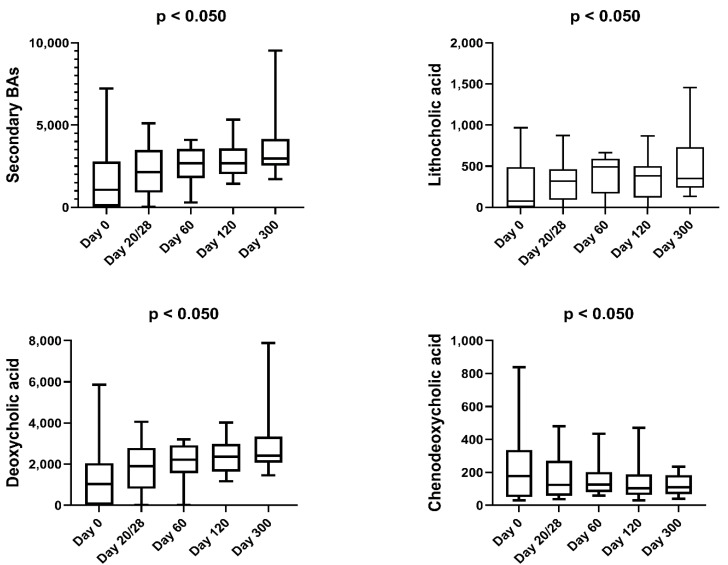
Fecal concentrations of BAs that significantly changed in CON cats over the course of the study. Minimums and maximums are displayed.

Table 5 shows the complete list of fecal metabolites for which the abundances changed significantly within the first year of life in CON cats, presented as mean values with standard deviations. Within the CON cats, total secondary BAs (*p* = 0.003) as well as some individual secondary BAs (LA, *p* = 0.038; DCA, *p* < 0.050) increased, while some individual primary BAs (CDCA percentage, *p* = 0.006) decreased after 2 months of age (i.e., day 0) (Figure 7). The abundance of linoleic acid (*p* = 0.013), total sterols (*p* = 0.039), as well as some individual sterols (coprostanol, *p* = 0.017; campesterol, *p* = 0.012; stigmasterol, *p* = 0.001; fusosterol, *p* = 0.016; beta-sitosterol, *p* = 0.004; sitostanol, *p* < 0.001) significantly increased. 

#### 3.2.2. Amoxicillin/Clavulanic Acid Group 

**Figure 8:**
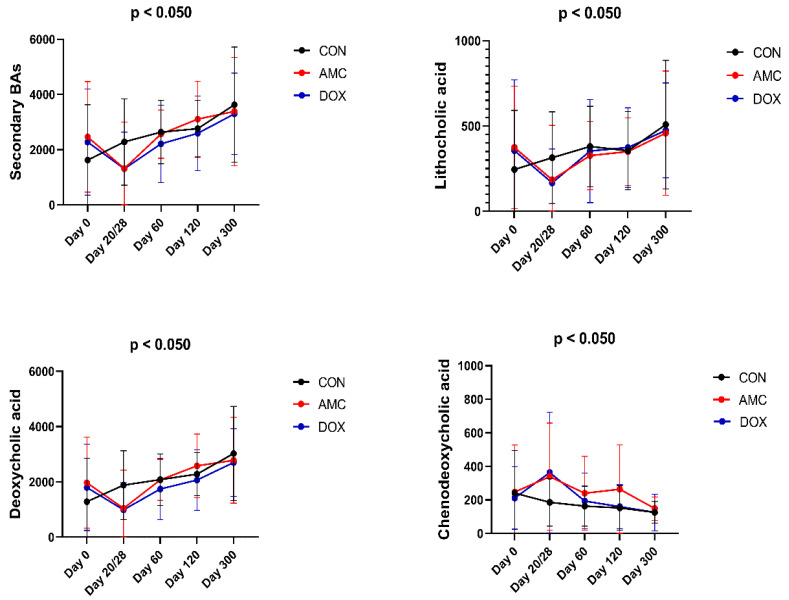
Fecal concentrations of BAs that significantly changed in CON cats over the course of the study. Means and standard deviations are displayed.

Fecal concentrations of total (*p* < 0.001) and individual secondary BAs (LA, *p* = 0.004; DCA, *p* = 0.010) increased, while individual primary BAs (CDCA percentage, *p* = 0.006) decreased after amoxicillin–clavulanic acid discontinuation (after the age of 3 months). In contrast to CON cats, where total secondary BAs increased and total primary BAs (CDCA percentage) decreased from 2 to 3 months of age, the age-related increase in secondary BAs and decrease in primary BAs was delayed by 1 month and started to appear after 3 months of age (Figure 8) in AMC cats. Individual fatty acids significantly decreased during treatment, as well as 1 month after the discontinuation of the antibiotic. Sterols changed over time and showed a similar pattern when compared to the pattern observed in CON cats (Table 6). 

#### 3.2.3. Doxycycline-Treated Cats 

**Figure 9:**
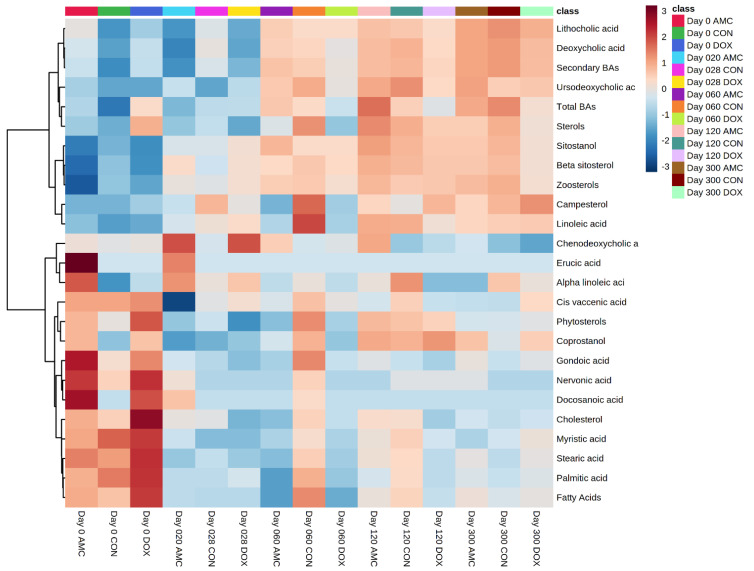
Heatmap clustering metabolites in feces of CON, AMC, and DOX cats over time (days 0, 20/28, 60, 120 300). For simplicity, the figure only displays the average concentrations of the top 25 metabolites that were different (*q* < 0.05) based on a liner mixed-effects analysis. Each row represents the average intensity of one metabolite for all three groups (CON, AMC, and DOX) and for all sampling times.

Similar to the AMC-treated cats, the fecal concentrations of total secondary bile acids (*p* < 0.050) and some individual secondary BAs (LA, *p* = 0.006; DCA, *p* < 0.050), as well as some individual primary BAs (CDCA percentage, *p* = 0.004), started to increase after the end of treatment with doxycycline (Figure 8). Individual fatty acids and sterols significantly decreased during doxycycline treatment (Table 7), which was not observed within the CON cats at this age. Heatmaps, which graphically represent variations in fecal metabolite concentrations within each group of cats, are presented in Figure 9. 

## 4. Discussion

This study showed that young cats treated with antibiotics had significant alterations of their serum and fecal metabolites compared to control cats. Amoxicillin/clavulanic acid and doxycycline caused perturbations in the pattern of concentrations of metabolites related to amino acids, carbohydrates, lipids, nucleotides, and bile acids that were not observed in control cats. 

Alanine, leucine, and valine were the most prevalent amino acids in the serum of cats regardless of age or antibiotic treatment. Most amino acid concentrations did not significantly change during the first year of age, nor with antibiotic treatment (valine, tyrosine, serine, proline, phenylalanine, methionine, lysine, leucine, histidine, glutamic acid, cysteine, asparagine, and alanine). The concentration of other amino acids did not change in CON cats and, therefore, were not significantly affected by aging, but changed within the antibiotic-treated cats (threonine and isoleucine), while others were affected both by age and by antibiotic treatment (glycine, tryptophan, glutamine, and aspartic acid). Particular GI microorganisms produce amino acids through the fermentation of carbon and nitrogen sources, or consume them as nutrients, or use them for toxin synthesis [49,50]. For example, *E. coli* can produce threonine and consume serine or cysteine [51]. A study in infants showed a profound impact of beta-lactams on fecal amino acid concentrations with reductions in fecal serine, tyrosine, and lysine [52]. Another study in rats showed a reduction in the serum concentrations of glutamine, serine, and glutamate during a 28-day course with tetracyclines [53]. Therefore, antibiotics might interfere with microbial amino acid metabolism by altering the composition of GI microbial members that are amino acid producers or amino acid consumers. Glycine declined significantly within the first year in CON cats, and this reduction might be attributed to its high requirement for muscle growth, an effect that has been reported also in children during their first year of age [54]. 

The pattern in serum concentrations of metabolites classified as antioxidants (trans-4-hydroxyproline, methionine sulfoxide, and indole-3-propionic acid) also differed between control cats and cats treated with antibiotics. Trans-4-hydroxyproline is a hydrolysate derivative of hydroxyproline, and is highly abundant in collagen [55]. Trans-4-hydroxyproline increased significantly over time in both antibiotic-treated groups, while it decreased in control cats after 3 months of age. The degradation of collagen can cause increased concentrations of trans-4-hydroxyproline and takes place during oxidative stress [56]. Methionine sulfoxide concentrations decreased in control cats, and no changes were observed in antibiotic-treated cats. Methionine sulfoxide is formed by the oxidation of methionine in the presence of (ROS), and is also a potential marker of oxidative stress [57]. Trans-4-hydroxyproline and methionine sulfoxide serum concentrations are altered in horses with resistance to insulin [58] and obese mice [59]. The development of an oxidative stress state is a common effect of antibiotic treatment. Bactericidal (e.g., ampicillin, norfloxacin, gentamicin, and rifampin) antibiotic-induced alterations in mitochondrial morphology and function lead to the accumulation of reactive oxygen species (ROS) within the bacterial cell, which eventually leads to its disruption [60]. It is also important to mention that not only is it bacterial cells that undergo oxidative stress during treatment with bactericidal antibiotics, but also eukaryotic cells [61]. Indole-3-propionic acid was detected at significantly lower concentrations in cats treated with amoxicillin/clavulanic acid, compared to control cats on the last day of treatment. Indole-3-propionic acid is a tryptophan-derived microbial metabolite that exerts a protective role on the intestinal mucosal barrier through increasing mucin synthesis, and with other beneficial products produced by the intestinal goblet cells [62]. The reduced indole-3-propionic acid concentrations could also explain the increased serum concentrations of tryptophan in AMC cats at the end of treatment. Reduced indole 3-propionic acid concentrations have been found in mice treated with neomycin, which was significantly associated with weight gain. Exogenous indole-3-propionic acid administration led to a two-fold reduction in body weight gain in mice treated with neomycin compared to mice treated with neomycin alone, suggesting a potential link of antibiotic-induced alteration in the microbial metabolism of tryptophan and obesity [63]. 

Concentrations of purines (guanine) and pyrimidines (uracil, thymidine, and pseudo-uridine) significantly increased during treatment with doxycycline, and this might explain the reduced serum concentrations of aspartate, which is deaminated in the GI tract for the production of purines and pyrimidines. Nucleotide (purines and pyrimidines) biosynthesis pathways are suggested to be involved in antibiotic lethality. Bacterial nucleotide biosynthesis is stimulated during antibiotic treatment [64,65]. The synthesis of nucleotides for ATP storage requires more energy, followed by increasing cellular respiration, central carbon metabolism, and oxidative stress, and these metabolic disturbances eventually lead to bacterial cell death [39]. However, this mechanism has been described with bactericidal and not with bacteriostatic antibiotics, such as doxycycline. Therefore, these data may suggest that doxycycline may alter bacterial nucleotide metabolism, an effect that was not observed with amoxicillin treatment.

Cats treated with antibiotics also showed a different pattern in concentrations of the metabolites classified as carbohydrates or as derivatives of carbohydrate metabolism (sugar phosphates, deoxy and amino sugars, sugar alcohols, sugar acids, and inositol phosphate). Of the monosaccharides, serum glycose and fucose concentrations increased over time in AMC cats, and fructose concentrations increased in DOX cats. Monosaccharides can be utilized by the GI microbiota, and their consumption results in an increase in bacterial species that are beneficial for the host, such as *Bifidobacterium* spp. and *Bacteroides* spp. [66]. Therefore, the increased serum concentrations of fucose, fructose, and glycose might be attributed to an antibiotic-induced reduction of beneficial microbial species with the ability to ferment monosaccharides. Among the serum sugar alcohols, erythritol and arabitol decreased in control cats, while myoinositol concentration decreased in doxycycline-treated cats. Increased concentrations of serum sugar alcohols have been associated with disturbances in colonization resistance and susceptibility to infection with intestinal pathogens [67,68], liver disease [69], and obesity [70]. Therefore, an age-related reduction of serum sugar alcohol concentrations might be a marker of gut health. Inositol-4-monophosphate concentration decreased in AMC cats at the end of treatment, compared to the CON cats. Inositol and its phosphates play multiple beneficial roles for the host, including serving as co-factors for mRNA expression, maintaining phosphate homeostasis, and are involved in insulin signaling [71]. A possible explanation for the reduction of inositol phosphate may be reduced intestinal absorption, a process known to be orchestrated by the GI microbiome. It has been reported that a dysbiotic microbiome can reduce the absorption of inositol and its phosphates [72].

Serum arachidonic acid decreased in both antibiotic-treated groups during treatment, before reaching similar concentrations in control cats. Arachidonic acid is an omega-6 fatty acid and is the precursor of inflammatory mediators, namely the eicosanoids. Eicosanoids are a family of signaling molecules that include prostaglandins and leukotrienes [29]. In cats with inflammatory bowel disease (IBD), it has been suggested that large amounts of omega-6 fatty acids in the diet should be avoided, and instead be replaced with omega-3 fatty acids to avoid inflammation [73]. Many drugs target arachidonic acid metabolism [29]. For example, it has been shown in vitro that beta-lactams can suppress arachidonic acid release from platelets to form thromboxanes [74]. In another study, a combination of ampicillin and chloramphenicol increased eicosanoid concentrations [75]. Therefore, both antibiotics might have an impact on eicosanoid formation. Alternatively, some bacterial members are reported to be able to metabolize arachidonic acid. Serum arachidonic acid reduction during antibiotic treatment in our study might reflect an increase in bacteria that are able to metabolize it [76]. 

The concentration of both total and some individual secondary BAs significantly increased after the age of 2 months in the feces of CON cats. In one study, 9-week-old puppies had lower concentrations of fecal secondary BAs compared to 1-year-old dogs [77]. Therefore, similarly to young dogs, microbial converters of primary to secondary BAs in 2-month-old cats have not yet reached an adult plateau. In contrast, in both groups treated with antibiotics, the increase in secondary BAs appeared after the age of 3 months, i.e., after antibiotic discontinuation. This suggests that antibiotics caused a transient suppression of microbial bile acid converters. Secondary BAs are commonly decreased after antibiotic treatment in rodents [78], humans [79], adult dogs [36], and cats [31,32]. Mounting evidence suggests that secondary BAs can suppress the proliferation of GI pathogens and maintain colonization resistance [80]. Secondary BA have direct antimicrobial effects and can indirectly stimulate the production of antimicrobial peptides after interaction with the farnesoid X receptor (FXR) [80]. During immune system maturation, the microbiome also matures and informs the immune system about which bacteria can be considered to be harmful or pathogenic [81,82,83]. A reduction in secondary BAs at this critical developmental window of cats could therefore allow the proliferation of GI pathogens and engender a lack of a proper immune response due to the immune system immaturity at this age. 

Fecal concentrations of cholesterol did not change within AMC or CON cats, but in DOX cats, cholesterol concentrations decreased significantly and remained decreased for 3 months after doxycycline withdrawal. Several possible mechanisms could lead to reduced concentrations of cholesterol in the feces of DOX cats. Cholesterol can be metabolized by bacteria within the large intestine, mainly producing the poorly absorbable compound coprostanol [84]. However, fecal coprostanol concentrations remained unchanged in DOX cats; moreover, in humans, the bacterial degradation of cholesterol to cholestanol starts after the age of 6 months [85,86]. Therefore, a doxycycline-induced decrease in the amount of coprostanol-producing bacteria could not explain the reduced fecal cholesterol concentrations identified in this study. The depletion of the GI microbiota by antibiotics is documented to increase the intestinal absorption of cholesterol, which could potentially explain the reduced concentrations of cholesterol in feces in DOX cats; yet this was not observed in AMC cats [87]. Lathosterol is a precursor of the endogenous cholesterol biosynthesis pathway, and this was also only reduced in DOX cats, suggesting a suppression of cholesterol biosynthesis and a secondary reduction of fecal cholesterol concentrations [88]. In humans, it has been shown that particular antibiotics, including metronidazole, can have lipid-lowering effects [89]. 

Interestingly, serum concentrations of some metabolites (15/185) were differentially abundant among the groups before exposure to antibiotics. The exact reason for these differences among groups is unknown. It could be considered that a URTI could have affected the GI microbiota in these cats. However, an acute URTI is a condition that is typically localized in the upper respiratory tract, and there is no evidence that it alters the composition of the GI microbiome or metabolite concentrations in cats. In addition, cats in the control group also had mild signs of URTI that did not require antibiotic treatment. Therefore, baseline differences in serum metabolic profiles are most likely attributable to individualized differences driven by the immaturity of the microbiome at this age, rather than URTI. In humans, individual differences in microbial and metabolic profiles have been identified before the microbiome reaches maturity, i.e., around 1 to 4 years of age [90]. Another potential limitation of the study that could at least partially explain the interindividual differences observed among the study groups is the small numbers of cats enrolled. However, previous studies applying similar methodologies have used similar numbers of cats [20,31,32]. Finally, the dose used for amoxicillin/clavulanic acid was higher (20 mg/kg) than the one recommended for upper respiratory tract infection in cats (11–13 mg/kg), but still within the normal range for amoxicillin/clavulanic acid. It is currently unknown whether a higher dose of antibiotics could be associated with a more profound impact on the metabolomic profiles of cats.

In summary, both antibiotics temporarily affected concentrations of serum and fecal metabolites in young cats, but these changes were no longer present at 10 months after antibiotic therapy. The long-term clinical consequences of these disturbances remain unknown. Further studies are required to investigate the impact of these changes on potential disease susceptibility and proneness to pathogen colonization in cats. 

## 5. Conclusions

Both amoxicillin and doxycycline affected the concentrations of several fecal and serum metabolites during treatment. However, these changes were transient and did not persist at 10 months after the withdrawal of the antibiotics. Cats treated with amoxicillin/clavulanic acid for 20 days showed reduced serum concentrations of inositol-4-monophosphate, and increased serum concentrations of indole-3-propionic acid on the last day of treatment compared to control cats. Cats treated with doxycycline for 28 days showed increased serum concentrations of uracil, hypoxanthine, and guanine on the last day of treatment compared to control cats. Fecal nervonic acid was detected at significantly lower concentrations in both antibiotic-treated groups on the last day of treatment, and this effect lasted for 3 months after antibiotic withdrawal. Fecal cholesterol was also persistently reduced in doxycycline-treated cats compared to control cats for 3 months after doxycycline discontinuation. When the pattern in the concentrations of metabolites was compared within each group, amino acids, antioxidants, metabolites related to carbohydrate metabolism, bile acids, sterols, and fatty acids were differentially expressed over time in each group. Similar to the developing microbiome, the concentrations of serum and fecal metabolites are characterized by high interindividual variability at 2 months of age in cats, and antibiotics appeared to delay the maturation of the serum and fecal metabolome. Further studies are required to investigate a potential association between the changes in these metabolites and potential disease susceptibility later in life.

## Figures and Tables

**Table 1:** Peak heights of serum metabolites that significantly differed among the groups at each timepoint.

Metabolite	Classification	AMC Group	CON Group	DOX Group
Day 0				
Glutamine	Amino acid	552,948 ^a^ ± 263,874	872,940 ^a^ ± 336,986	735,477 ± 296,472
Asparagine	Amino acid	28,024 ^a,b^ ± 8064	39,820 ^a^ ± 14,906	37,632 ^b^ ± 9713
Alanine	Amino acid	1,571,042 ^a^ ± 777,177	2,077,072 ^a^ ± 473,101	1,921,038 ± 581,830
Citric acid	Organic acid	257,011 ^a,b^ ± 124,738	332,840 ^a^ ± 112,121	346,138 ^b^ ± 99,573
Trans-4- hydroxyproline	Antioxidant	255,037 ^a^ ± 192,296	362,099 ^a^ ± 180,627	256,459 ± 126,726
Ribose	Sugar	17,584 ± 10,043	21,027 ^a^ ± 12,089	31,123 ^a^ ± 16,217
Threitol	Sugar alcohol	6119 ^a^ ± 8254	1801 ^a^ ± 585	2897 ± 2568
3-hydroxybutyric acid	Fatty acid	1,192,909 ^a^ ± 2,274,124	112,391 ^a^ ± 80,512	311,592 ± 407,090
Beta-sitosterol	Sterol	2458 ^a^ ± 1340	4459 ^a^ ± 2159	2766 ± 1602
Dehydrocholesterol	Zoosterol	2775 ^a^ ± 1484	5023 ^a^ ± 3152	3991 ± 1571
Phenylethylamine	Polyamine	86,072 ± 12,733	49,633 ^a^ ± 59,675	28,350 ^a^ ± 23,642
Glyceric acid	Trionic acid	27,312 ^a^ ± 11,511	18,797 ^a,b^ ± 5325	31,637 ^b^ ± 16,222
2-hydroxyglutaric acid	Glutaric acid	9528 ^a^ ± 4490	12,753 ^a^ ± 5398	10,048 ± 3521
2-hydroxybutanoic acid	Hydroxybutyric acid	193,658 ^a^ ± 170,262	93,973 ^a^ ± 61,660	123,541 ± 69,982
2-deoxypentitol	Tetrol	3796 ^a,b^ ± 5408	1187 ^a^ ± 292	1388 ^b^ ± 404
Day 20/28				
Uracil	Pyrimidine	6180 ± 2823	4458 ^a^ ± 1647	8912 ^a^ ± 4261
Hypoxanthine	Purine	84,182 ± 35,210	58,815 ^a^ ± 23,693	94,958 ^a^ ± 28,239
Guanine	Purine	3031 ± 1878	2071 ^a^ ± 946	3936 ^a^ ± 2018
Inositol-4- monophosphate	Inositol phosphate	1289 ^a,b^ ± 466	2072 ^a^ ± 653	2058 ^b^ ± 872
Indole-3- propionic acid	Antioxidant	2624 ^a,b^ ± 3382	8815 ^a^ ± 3066	7491 ^b^ ± 3823
Glycolic acid	Lipid	16,124 ^a^ ± 2687	17,618 ± 4914	27,515 ^a^ ± 19,908

Values are mean ± SD; ^a,b^ indicate significant differences between groups after FDR adjustment. Values with different superscripts (^a,b^) in the same row are significantly different (*p* < 0.05).

**Table 2:** Peak heights of serum metabolites that differed significantly over time within CON cats.

Metabolites	Classification	Day 0	Day 28	Day 300
Tryptophan	Amino acid	255,630 ^a^ ± 96,825	344,259 ± 139,221	441,120 ^a^ ± 137,367
Glycine	Amino acid	883,554 ± 378,246	887,547 ^b^ ± 233,365	546,185 ^b^ ± 165,401
4-hydroxyphenylacetic Acid	Acetic acid	3015 ^b^ ± 1602	2829 ± 2052	1252 ^b^ ± 525
2-hydroxyglutaric Acid	Glutaric acid	12,753 ^c^ ± 5398	10,301 ^d^ ± 3394	4327 ^c,d^ ± 906
Pseudo uridine	Pyrimidine	15,377 ± 6106	17,688 ^c^ ± 2482	12,642 ^c^ ± 3254
Trans-4- Hydroxyproline	Antioxidant	362,099 ± 180,627	463,524 ^b^ ± 154,783	229,341 ^b^ ± 113,943
Methionine sulfoxide	Antioxidant	107,175 ^b^ ± 52,419	93,536 ± 35,520	59,856 ^b^ ± 33,237
Maltose	Sugar	15,491 ^b^ ± 7688	10,346 ± 4911	6515 ^b^ ± 2847
Erythritol	Sugar alcohol	12,511 ^a^ ± 4017	12,764 ± 1622	1048 ^a^ ± 1623
Arabitol	Sugar alcohol	9453 ± 3557	13,775 ^c^ ± 3221	8836 ^c^ ± 2273
Threonic acid	Sugar acid	31,888 ^a^ ± 21,050	16,298 ± 9022	13,589 ^a^ ± 7100
Putrescine	Polyamine	8201 ^c^ ± 2687	7366 ± 3102	5258 ^c^ ± 1630
Phenylethylamine	Polyamine	49,633 ± 59,675	31,497 ^a^ ± 119,931	11,004 ^a^ ± 8318
Serotonin	Hormone	83,366 ^c^ ± 36,347	85,772 ± 40,067	54,524 ^c^ ± 32,985
Phosphate	Ester	658,084 ^b^ ± 154,258	740,668 ^a^ ± 228,235	474,287 ^a,b^ ± 128,407
2-hydroxy- 2-methylbutanoic acid	Organic acid	11,851 ^a^ ± 5531	9429 ± 3784	6686 ^a^ ± 1321
Creatinine		107,839 ^b^ ± 52,332	119,931 ± 39,720	188,484 ^b^ ± 41,887

Values are mean ± SD; ^a,b,c,d^ indicate significant differences over time in AMC cats after Bonferroni correction (^a^ 0.025 < *p* < 0.050; ^b^ 0.005 ≤ *p* < 0.025; ^c^ 0.005 ≤ *p* < 0.001; ^d^ *p* ≤ 0.001).

**Table 3:** Peak heights of serum metabolites that significantly differed over time in cats of the AMC group.

Metabolites	Classification	Day 0	Day 20	Day 300
Tryptophan	Amino acid	250,388 ^d^ ± 119,739	389,432 ^c,d^ ± 66,866	486,581 ^d^ ± 95,354
Threonine	Amino acid	200,577 ^a^ ± 108,054	317,721 ± 255,426	277,046 ^a^ ± 109,534
Oxoproline	Amino acid	721,708 ^a^ ± 174,709	815,968 ± 134,778	981,791 ^a^ ± 184,254
Isoleucine	Amino acid	514,101 ± 188,354	503,052 ^b^ ± 83,031	404,230 ^b^ ± 72,426
Tartaric acid	Organic acid	455 ± 348	380 ^b^ ± 139	1108 ^b^ ± 706 ± 882
Aconitic acid	Organic acid	2615 ^a^ ± 1457	1594 ^a^ ± 837	1614
2-hydroxyglutaric acid	Glutaric acid	9528 ^c^ ± 4490	12,206 ^d^ ± 3939	4244 ^c,d^ ± 1019
Trans-4-hydroxyproline	Antioxidant	255,037 ^b^ ± 192,296	460,027 ^b^ ± 112,402	291,014 ± 128,520
Glucose	Sugar	3,145,429 ± 643,471	3,215,996 ^b^ ± 497,544	4,251,770 ^b^ ± 1,155,169
Fucose	Sugar	7136 ± 1408	7344 ^a^ ± 1362	9371 ^a^ ± 2347
Phosphate	Ester	585,752 ± 180,423	724,383 ^d^ ± 135,884	461,411 ^d^ ± 112,401
Beta-sitosterol	Sterol	2458 ^c^ ± 1340	5775 ± 3217	6342 ^c^ ± 3243
Arachidonic acid	Fatty acid	20,304 ^b^ ± 7954	12,796 ^a,b^ ± 4318	19,069 ^a^ ± 5576
5-aminovaleric acid	Fatty acid	27,554 ^a^ ± 25,615	12,374 ± 10,395	4658 ^a^ ± 2946
5-hydroxymethyl- 2-furoic acid	Furoic acid	5903 ± 9134	993 ^b^ ± 256	1571 ^b^ ± 388
Creatinine		169,183 ± 97,973	110,233 ^a^ ± 39,656	181,369 ^a^ ± 57,411

Values are mean ± SD; ^a,b,c,d^ indicate significant differences over time in AMC cats after Bonferroni correction (^a^ 0.025 < *p* < 0.050; ^b^ 0.005 ≤ *p* < 0.025; ^c^ 0.005 ≤ *p* < 0.001; ^d^ *p* ≤ 0.001).

**Table 4:** Peak heights of serum metabolites that significantly differed over time within cats of the DOX group.

Metabolites	Classification	Day 0	Day 28	Day 300
Tryptophan	Amino acid	267,090 ^d^ ± 124,026	393,276 ± 108,905	534,822 ^d^ ± 126,493
Threonine	Amino acid	155,280 ^d^ ± 71,885	233,122 ± 125,192	232,976 ^d^ ± 55,123
Glutamine	Amino acid	735,477 ± 296,473	685,995 ^a^ ± 270,345	1,058,116 ^a^ ± 241,969
Aspartic acid	Amino acid	26,034 ± 7550	30,308 ^a^ ± 6213	22,840 ^a^ ± 4077
2-hydroxyglutaric acid	Glutaric acid	10,048 ^c^ ± 3521	12,499 ^d^ ± 3377	4425 ^c,d^ ± 1062
Uracil	Pyrimidine	6916 ^a^ ± 3400	8912 ^c^ ± 4261	3795 ^a,c^ ± 1863
Thymidine	Pyrimidine	30,479 ^a^ ± 11403	47,596 ^a^ ± 16,194	36,446 ± 8196
Pseudo uridine	Pyrimidine	18,234 ^a^ ± 4993	18,167 ± 7313	13,255 ^a^ ± 2608
Hypoxanthine	Purine	74,971 ± 24,171	94,958 ^a^ ± 28,239	61,218 ^a^ ± 23,140
Trans-4-hydroxyproline	Antioxidant	256,459 ^b,c^ ± 126,726	483,290 ^c^ ± 125,486	290,840 ^b^ ± 112,972
Fructose	Sugar	270,389 ^a^ ± 100,509	253,463 ± 69,322	446,898 ^a^ ± 206,582
Myo-inositol	Sugar alcohol	328,115 ± 196,034	404,677 ^a^ ± 133,018	265,394 ^a^ ± 128,091
Isothreonic acid	Sugar acid	12,508 ^c^ ± 3518	12,359 ^b^ ± 3379	7674 ^c,b^ ± 1083
Gluconic acid	Sugar acid	104,628 ^a^ ± 61,378	90,441 ± 47,982	43,425 ^a^ ± 43,335
Tocopherol alpha	Vitamin	41,000 ^a^ ± 22,358	20,256 ^a^ ± 8868	26,884 ± 12,928
Phosphate	Ester	646,227 ± 185,512	803,778 ^b^ ± 194,685	506,265 ^b^ ± 107,415
Lanosterol	Lanosterol	372 ± 180	257 ^a^ ± 60	344 ^a^ ± 66
Beta-sitosterol	Sterol	2766 ^a,b^ ± 1602	6238 ^a^ ± 2606	5392 ^b^ ± 2244
Arachidonic acid	Fatty acid	21,507 ± 8645	15,391 ^a^ ± 4627	21,035 ^a^ ± 4419
5-hydroxymethyl- 2-furoic acid	Furoic acid	1799 ± 1639	980 ^d^ ± 267	1628 ^d^ ± 417
Creatinine		144,622 ± 108,647	117,559 ^d^ ± 42,094	207,931 ^d^ ± 51,552

Values are mean ± SD; ^a,b,c,d^ indicate significant differences over time in DOX cats after Bonferroni correction (^a^ 0.025 < *p* < 0.050; ^b^ 0.005 ≤ *p* < 0.025; ^c^ 0.005 ≤ *p* < 0.001; ^d^ *p* ≤ 0.001).

**Table 5:** Concentrations of fecal metabolites that significantly differed over time within CON cats.

Metabolites	Classification	Day 0	Day 28	Day 60	Day 120	Day 300
Lithocholic acid	BA	244 ^a^ ± 346	313 ± 268	380 ± 236	355 ± 229	507 ^a^ ± 377
Deoxycholic acid	BA	1282 ^a,d^ ± 1576	1882 ± 1245	2082 ± 934	2281 ^a^ ± 781	3025 ^d^ ± 1705
Linoleic acid	Fatty Acid	6.54 ^b^ ± 3.87	7.92 ± 3.81	10.20 ^b^ ± 4.80	9.08 ± 4.69	7.95 ± 2.90
Coprostanol	Sterol	0.17 ^a,b,c^ ± 0.09	0.43 ± 0.75	0.91 ^c^ ± 1.21	0.90 ^a^ ± 1.31	0.68 ^b^ ± 0.93
Campesterol	Sterol	0.24 ^b^ ± 0.15	0.34 ± 0.17	0.42 ^b^ ± 0.19	0.38 ± 0.15	0.42 ± 0.17
Stigmasterol	Sterol	0.12 ^b,c,d^ ± 0.07	0.17 ± 0.08	0.20 ^c^ ± 0.06	0.20 ^b^ ± 0.09	0.21 ^d^ ± 0.05
Fucosterol	Sterol	0.03 ^a,c^ ± 0.04	0.06 ± 0.04	0.07 ^a^ ± 0.04	0.06 ± 0.04	0.07 ^c^ ± 0.04
Beta-sitosterol	Sterol	0.66 ^c^ ± 0.38	0.92 ± 0.47	1.05 ± 0.43	0.97 ± 0.40	1.17 ^c^ ± 0.43
Sitostanol	Sterol	0.37 ^b,c,d^ ± 0.27	0.63 ± 0.33	0.78 ^b^ ± 0.28	0.80 ^c^ ± 0.30	0.88 ^d^ ± 0.27
Secondary BAs	BA	1622 ^a,c^ ± 2003	2282 ± 1566	2636 ^a^ ± 1147	2761 ± 1027	3629 ^c^ ± 2086
Chenodeoxycholic acid (%)	BA	13.03 ^b^ ± 15.78	7.07 ± 5.70	5.06 ± 3.19	3.95 ± 1.78	3.52 ^b^ ± 2.15
Ursodeoxycholic acid (%)	BA	2.42 ^a^ ± 3.45	2.60 ± 2.87	4.94 ^a^ ± 4.05	3.62 ± 2.36	2.58 ± 1.64
Zoosterols	Sterol	1.42 ^b,c,d^ ± 0.86	2.13 ± 1.08	2.53 ^c^ ± 0.97	2.41 ^b^ ± 0.90	2.76 ^d^ ± 0.94
Sterols	Sterol	3.83 ^a^ ± 1.76	4.26 ± 1.57	5.63 ^a^ ± 2.20	5.12 ± 1.80	4.98 ± 1.43

Values are mean ± SD; ^a,b,c,d^ indicate significant differences over time in CON cats after Bonferroni correction (^a^ 0.025 < *p* < 0.050; ^b^ 0.005 ≤ *p* < 0.025; ^c^ 0.005 ≤ *p* < 0.001; ^d^ *p* ≤ 0.001).

**Table 6:** Concentrations of fecal metabolites that significantly differed over time within AMC cats.

Metabolites	Classification	Day 0	Day 20	Day 60	Day 120	Day 300
Lithocholic acid	BA	374 ± 359	184 ^c^ ± 319	325 ± 200	349 ± 198	458 ^c^ ± 364
Deoxycholic acid	BA	1968 ± 1652	1041 ^b,c,d^ ± 1392	2073 ^b^ ± 742	2584 ^c^ ± 1151	2783 ^d^ ± 1549
Palmitic acid	Fatty acid	12.61 ^b^ ± 5.95	9.15 ± 3.73	7.49 ^b^ ± 3.11	10.00 ± 4.90	9.81 ± 4.16
Stearic acid	Fatty acid	9.94 ^c,d^ ± 6.35	4.20 ^d^ ± 1.20	4.17 ^c^ ± 1.60	6.31 ± 4.34	6.29 ± 4.58
Nervonic acid	Fatty acid	0.49 ^a^ ± 0.62	0.28 ± 0.46	0.16 ^a^ ± 0.05	0.19 ± 0.09	0.20 ± 0.13
Campesterol	Sterol	0.19 ^a,c,d^ ± 0.11	0.30 ± 0.15	0.34 ^a^ ± 0.15	0.38 ^c^ ± 0.14	0.38 ^d^ ± 0.13
Stigmasterol	Sterol	0.11 ^a,c,d^ ± 0.07	0.18 ± 0.08	0.19 ^a^ ± 0.06	0.21 ^d^ ± 0.09	0.20 ^c^ ± 0.05
Fucosterol	Sterol	0.02 ^a^ ± 0.03	0.06 ± 0.06	0.10 ^a^ ± 0.10	0.07 ± 0.05	0.07 ± 0.03
Beta-sitosterol	Sterol	0.49 ^b,c,d^ ± 0.35	0.92 ± 0.53	1.00 ^b^ ± 0.42	1.04 ^d^ ± 0.45	1.03 ^c^ ± 0.35
Sitostanol	Sterol	0.29 ^a,d^ ± 0.33	0.61 ^a^ ± 0.34	0.77 ^d^ ± 0.19	0.82 ^d^ ± 0.25	0.81 ± 0.33
Secondary BA	BA	2463 ± 2002	1316 ^c,d^ ± 1688	2565 ^c^ ± 878	3109 ^d^ ± 1362	3387 ^d^ ± 1964
Chenodeoxycholic acid (%)	BA	9.15 ± 7.95	13.83 ^b^ ± 11.72	6.86 ± 5.35	6.31 ± 5.92	4.59 ^b^ ± 3.19
Zoosterols	Sterol	1.10 ^c,d^ ± 0.86	2.07 ^c^ ± 1.13	2.38 ^d^ ± 0.85	2.52 ^d^ ± 0.90	2.49 ± 0.81

Values are mean ± SD; ^a,b,c,d^ indicate significant differences over time in AMC cats after Bonferroni correction (^a^ 0.025 < *p* < 0.050; ^b^ 0.005 ≤ *p* < 0.025; ^c^ 0.005 ≤ *p* < 0.001; ^d^ *p* ≤ 0.001).

**Table 7:** Concentrations of fecal metabolites that significantly differed over time within DOX cats.

Metabolites	Classification	Day 0	Day 28	Day 60	Day 120	Day 300
Lithocholic acid	BA	355 ± 415	166 ^b^ ± 198	352 ± 302	373 ± 233	474 ^b^ ± 278
Deoxycholic acid	BA	1798 ± 1562	985 ^a,d^ ± 988	1740 ± 1110	2065 ^a^ ± 1092	2702 ^d^ ± 1219
Myristic acid	Fatty acid	0.54 ^a,d^ ± 0.28	0.25 ^d^ ± 0.07	0.25 ^d^ ± 0.09	0.32 ^a^ ± 0.15	0.39 ± 0.25
Palmitic acid	Fatty acid	14.65 ^b,c,d^ ± 4.60	9.61 ^b^ ± 4.35	8.49 ^d^ ± 4.17	8.87 ^c^ ± 2.72	10.69 ± 5.05
Stearic acid	Fatty acid	11.73 ^c,d^ ± 6.10± 6.10	4.34 ^d^ ± 1.67	4.71 ^d^ ± 2.67	4.93 ^c^ ± 2.01	6.25 ± 3.80
Docosanoic acid	Fatty acid	0.29 ^a,c,d^ ± 0.21	0.12 ^d^ ± 0.03	0.13 ^c^ ± 0.04	0.16 ^a^ ± 0.08	0.16 ± 0.07
Gondoic acid	Fatty acid	0.60 ^a,b^ ± 0.44	0.26 ^b^ ± 0.08	0.27 ^a^ ± 0.14	0.33 ± 0.17	0.37 ± 0.25
Nervonic acid	Fatty acid	0.45 ^b,c,d^ ± 0.38	0.15 ^d^ ± 0.05	0.17 ^c^ ± 0.06	0.22 ± 0.11	0.21 ^b^ ± 0.09
Cholesterol	Sterol	3.15 ^b,c,d^ ± 2.28	1.05 ^d^ ± 0.47	1.26 ^c^ ± 0.69	1.40 ^c^ ± 0.77	1.51 ^b^ ± 0.58
Lathosterol	Sterol	0.02 ^a,b^ ± 0.00	0.02 ^a^ ± 0.00	0.02 ^b^ ± 0.00	0.02 ± 0.00	0.02 ± 0.00
Sitostanol	Sterol	0.34 ^b,c^ ± 0.37	0.75 ± 0.43	0.63 ± 0.24	0.83 ^b^ ± 0.31	0.82 ^c^ ± 0.50
Secondary ΒA	BA	2277 ± 1919	1307 ^a,d^ ± 1322	2212 ± 1405	2592 ^a^ ± 1352	3299 ^d^ ± 1471
Secondary BA (%)	BA	64.10 ± 40.69	49.94 ^a^ ± 38.40	76.56 ± 31.54	79.12 ± 27.13	84.76 ^a^ ± 20.83
Primary BA (%)	BA	35.90 ± 40.69	50.06 ^a^ ± 38.40	23.44 ± 31.54	20.88 ± 27.13	15.24 ^a^ ± 20.83
Chenodeoxycholic acid (%)	BA	6.84 ± 5.30	10.43 ^c^ ± 5.56	6.43 ± 4.08	5.80 ± 4.85	4.77 ^c^ ± 6.34
Deoxycholic acid (%)	BA	52.07 ± 35.48	39.43 ^b^ ± 33.36	60.77 ± 26.68	63.05 ± 22.05	69.04 ^b^ ± 19.09
Phytosterols	Sterol	4.02 ^b,c^ ± 2.78	1.57 ^c^ ± 0.74	1.78 ^b^ ± 0.73	2.48 ± 1.43	2.49 ± 1.41
Zoosterols	Sterol	1.37 ^a^ ± 0.97	2.40 ± 1.31	2.07 ± 0.74	2.56 ^a^ ± 0.86	2.52 ^a^ ± 1.34

Values are mean ± SD; ^a,b,c,d^ indicate significant differences over time in DOX cats after Bonferroni correction (^a^ 0.025 < *p* < 0.050; ^b^ 0.005 ≤ *p* < 0.025; ^c^ 0.005 ≤ *p* < 0.001; ^d^ *p* ≤ 0.001).

## Data Availability

Metabolomics raw data are available in supplementary files Tables S1 and S3.

## Supplementary Materials

The following are available online at https://www.mdpi.com/article/10.3390/ani12030330/s1, Table S1: List of raw metabolites identified in serum samples by untargeted metabolomics. Table S2: List of metabolites identified in serum samples by untargeted metabolomics. Mean and standard deviation by group and time point. Table S3: List of raw metabolites identified in fecal samples by targeted metabolomics. Table S4: List of metabolites identified in fecal samples by targeted metabolomics. Mean and standard deviation by group and time point.
